# Circulating Anti-GB3 Antibody as a Biomarker of Myocardial Inflammation in Patients with Fabry Disease Cardiomyopathy

**DOI:** 10.3390/jcm12124068

**Published:** 2023-06-15

**Authors:** Andrea Frustaci, Romina Verardo, Michele Magnocavallo, Rossella Scialla, Luigi Sansone, Matteo Antonio Russo

**Affiliations:** 1 IRCCS San Raffaele Rome, 00166 Roma, Italy; 2 Cellular and Molecular Cardiology Lab, IRCCS L. Spallanzani, 00149 Roma, Italy; 3 Arrhythmology Unit, Ospedale Fatebenefratelli Isola Tiberina—Gemelli Isola, 00186 Rome, Italy; 4 MEBIC Consortium, San Raffaele Open University, 00166 Rome, Italy

**Keywords:** Fabry disease, myocarditis, globotriaosylceramide, inflammation, cardiac biopsy

## Abstract

Background: Fabry disease cardiomyopathy (FDCM) has manifested some resistance to enzyme replacement therapy (ERT), particularly in its advanced phase. Recently, myocardial inflammation of autoimmune origin has been demonstrated in FDCM. Aims: The objective of this study was the assessment of circulating anti-globotriaosylceramide (GB3) antibodies as potential biomarkers of myocardial inflammation in FDCM, defined by the additional presence of ≥CD3+ 7 T lymphocytes/low-power field associated with focal necrosis of adjacent myocytes. Its sensitivity was based on the evidence of overlapping myocarditis at left ventricular endomyocardial biopsy. Methods and Results: From January 1996 to December 2021, 85 patients received a histological diagnosis of FDCM in our department and 48 (56.5%) of them had an overlapping myocardial inflammation with negative PCR for common cardiotropic viruses, positive antiheart, and antimyosin abs. The presence of anti-GB3 antibodies was evaluated with an in-house ELISA assay (BioGeM scarl Medical Investigational Research, MIR—Ariano Irpino, Italy), along with antiheart and antimyosin abs, in the FDCM patients and compared with control healthy individuals. The correlation between levels of circulating anti-GB3 autoantibody myocardial inflammation and FDCM severity was assessed. Anti-Gb3 antibodies were above the positivity cut-off in 87.5% of FDCM subjects with myocarditis (42 out of 48), while 81.1% of FDCM patients without myocarditis were identified as negative for Gb3 antibodies. Positive anti-Gb3 abs correlated with positive antiheart and antimyosin abs. Conclusions: The present study suggests a potential positive role of anti-GB3 antibodies as a marker of overlapping cardiac inflammation in patients with FDCM.

## 1. Introduction

Fabry disease is an X-linked inborn error of glycosphingolipid catabolism caused by mutations in the α-galactosidase A gene encoding the lysosomal hydrolase alpha-galactosidase A (α-Gal A) [1,2]. The marked deficiency or absence of α-Gal A activity results in a systemic accumulation of globotriaosylceramide (GB3) and related glycosphingolipids within the lysosomes, particularly in microvascular endothelial cells, vascular smooth muscle cells, renal tubular cells, podocytes, and cardiomyocytes [3]. 

Fabry disease cardiomyopathy (FDCM) is a major determinant of patients’ survival, and its management actually represents a major therapeutic challenge. Indeed, the impact of enzyme replacement therapy (ERT) on FDCM is still controversial, and while there is agreement that early ERT administration, particularly in the pre-hypertrophic phase, prevents the progression of the disease, the advanced form is believed to be irreversible [4,5]. 

The mechanisms of resistance to ERT are, however, still unclear.

In Fabry disease, as in other lysosomal storage disorders, substrate deposits in lysosomes fuel multiple pathogenic cascades that ultimately lead to an inflammatory response [6]. This may be a causative factor in the progression of the underlying lysosomal disorder despite the initiation of ERT, and may be one possible explanation for the clinical failure of therapy. Recently, our group demonstrated that immune-mediated myocarditis can be histologically recognized in 56% of patients with Fabry disease cardiomyopathy and its incidence correlated with disease severity [7].

Further studies showed that inflammatory and cardiac remodeling biomarkers are elevated in the sera of patients with Fabry disease and positively correlated with Mainz Severity Score Index scores, left ventricular hypertrophy, late gadolinium enhancement at cardiac magnetic resonance, and renal dysfunction [8]. From a pathogenic point of view, GB3 and its metabolites (for example lysoGB3) bind to Toll-like receptor 4, activating nuclear factor kB and T lymphocytes with the subsequent production of proinflammatory cytokines, leading to a chronic inflammatory state and associated vasculopathy [9].

However, both TNF and IL-6 plasma levels are nonspecific markers that are elevated in several conditions of chronic heart failure and correlate with a decreasing functional status as well as with all mortality causes [10]. These observations underline the need for a sensitive and specific circulating biomarker of myocardial inflammation in order to halt—through the administration of low-dose immunosuppression—its deleterious effect on disease progression and ERT resistance and improve FDCM treatment. This aim appears even more important if we believe that other organs such as the kidneys, brain, nerves, and vessels may be involved in the same pathway and theoretically benefit from the same solutions.

## 2. Materials and Methods

### 2.1. Patient Population

We enrolled all the patients with biopsy-proven and genetical diagnosis of FDCM in the study. Circulating levels of anti-GB3 autoantibodies were compared between patients with the histological presence (group A) or absence (group B) of myocardial inflammation (negative PCR for the cardiotropic viruses and positive antiheart and antimyosin antibodies).

The study complies with the Declaration of Helsinki; the locally appointed ethics committee approved the research protocol and informed consent was obtained from all subjects.

#### 2.1.1. Cardiac Studies

In all patients, cardiac investigations included noninvasive (ECG, Holter monitoring, 2D-echocardiography, and CMR) and invasive (coronary, left ventricular angiography, and EMB) studies after receiving written informed consent. Our institution is a tertiary referring center for the diagnosis and treatment of cardiomyopathies, myocarditis, and heart failure and obtained permission from the local ethical committee (Sapienza University, Rome, Italy. Opinion number 2016-003014-28 (FARM12JCXN)). EMB is regularly performed in our institution whenever a symptomatic heart muscle disease remains undiagnosed by noninvasive procedures including echocardiography and cardiac magnetic resonance. Biopsy samples, 5–8 per patient, were cut and stored at −80 °C, or processed for histology, immunohistochemistry, and electron microscopy. Two frozen samples per patient were processed for real-time PCR for the most common cardiotropic viruses in case of observation of overlapping myocarditis at histology.

The study complies with the Declaration of Helsinki, the locally appointed ethics committee (opinion number 6/2019) approved the research protocol, and informed consent was obtained from all subjects. The patients were strongly motivated to clarify the origin of symptoms and gave their written consent for the procedure.

Pathologists, echocardiographers, and CMR investigators were blind to the patients’ clinical and genetic background. They were informed after their report.

#### 2.1.2. Cardiac Magnetic Resonance

CMR exams were performed on a 1.5 Tesla scanner (Avanto, Siemens, Munich, Germany). The CMR protocol included: (i) cine CMR sequence acquired during breath holds in the short-axis, 2-chamber, and 4-chamber; (ii) black blood T2-weighted short tau inversion recovery images on short-axis planes covering the entire left ventricle during 6 to 8 consecutive breath holds for myocardial edema detection; (iii) late gadolinium-enhanced imaging performed 15 min after injection of 0.2 nmol/kg of gadoterate meglumine and signal intensity value 2 SDs above the mean signal intensity of the remote normal myocardium were considered suggestive of myocardial fibrosis; (iv) native T1 mapping imaging was performed, when available, using the MOLLI sequence on three short-axis views (one basal and two midventricular); (v) a T2 map was obtained using a T2-prepared True-FISP prototype sequence producing 3 single-shot images with 3 different T2 pulse preparations. A nonrigid registration algorithm and the two-parametric automatic curve fitting were automatically applied to generate the map. CMR image analysis was performed as previously described, extending the analysis method for T1 maps also to T2 maps. In particular, the values of T1 and T2 global were defined as normal, reduced, or increased compared to a reference range developed on a multiage sample of 100 healthy subjects of both sexes (normal value nT1 < 970 ms, T2 > 49.7 ms). 

#### 2.1.3. Invasive and Endomyocardial Biopsy Studies

Cardiac catheterization with left ventricular and coronary angiography was obtained in all patients. EMB was performed in the septal–apical region of the left ventricle. Endomyocardial samples were blindly evaluated by the same pathologist. He was informed of clinical and genetic characteristics after morphological examination.

#### 2.1.4. Histology and Electron Microscopy

For histological analysis, the endomyocardial samples were fixed in 10% buffered formalin and paraffin-embedded. Five-micron-thick sections were stained with hematoxylin and eosin and Masson trichrome. For electron microscopy studies, additional samples were fixed in 2% glutaraldehyde in a 0.1 M phosphate buffer at pH 7.3, post-fixed in osmium tetroxide, and processed following a standard protocol for embedding in Epon resin. Ultrathin sections were stained with uranyl acetate substitute and lead hydroxide. 

Controls for the morphological study consisted of surgical LV endomyocardial biopsies from patients with mitral stenosis, that, although not obtained from healthy individuals, were derived from a chamber without overload and were considered the samples nearest to normal endomyocardial tissue.

#### 2.1.5. Immunohistochemistry 

The histological diagnosis of myocarditis was based on the evidence of leukocyte infiltrates (≥14 leukocytes/2 mm) associated with necrosis of the adjacent myocytes, according to the Dallas criteria confirmed by immunohistochemistry. In particular, for the phenotypic characterization of inflammatory infiltrates, immunohistochemistry was performed for CD3, CD20, CD43, CD45RO, and CD68 (all Dako, Carpinteria, CA, USA).

### 2.2. Serum Studies

#### 2.2.1. Antiheart Autoantibodies

Patient sera were tested for the presence of circulating cardiac autoantibodies using a standard indirect immunofluorescence technique with a substrate of 4 μm thick unfixed cryostat sections of human atrium and intercostal skeletal muscle of blood group O patients. Briefly, patient sera were diluted 1/10 in pH 7.2 phosphate-buffered saline solution, and fluorescein-isothiocyanate-labeled rabbit antihuman immunoglobulin was used at 1:100 dilution. The intensity of immunofluorescence of the positive control (known positive serum) at 1/40 dilution was used as the cutoff point for positivity. Omission of the patient serum and known negative serum was included in every assay (negative controls).

#### 2.2.2. ELISA Test for Antimyosin and Gb3 Antibodies

Patient sera were screened for the presence of Gb3 and myosin antibodies, detected by using a quantitative human MYO ELISA kit (Elabscience, Wuhan, China) and the semiquantitative ELISA for Anti-Gb3 ELISA. As controls, we used a pool of normal sera from participants matched with patients for age and sex.

For antimyosin Ab detection, standards and undiluted samples were added to the micro-ELISA plate wells and combined with the specific antibody. The optical density proportional to the concentration of human myosin was measured spectrophotometrically at a wavelength of 450 nm. The detection range of the ELISA kit is 1.56 to 100 ng/mL. The concentration in the samples was calculated by comparing the optical density of the samples with the standard curve using the software interface GraphExpert Professional 1.4.0 (Elabscience, Wuhan, China) and expressed as ng/mL. The positivity of serum abs for Gb3 was correlated with overimposition of myocarditis in our patient population of 85 patients with FDCM whose diagnosis was obtained with the assessment of pathogenic GLA gene mutations and detection of Gb3 accumulation at histology, immunohistochemistry, and ultrastructural examination of left ventricular endomyocardial biopsies.

#### 2.2.3. Development of the Indirect ELISA for the Detection of Anti-Gb3 Antibodies

**Figure 1:**
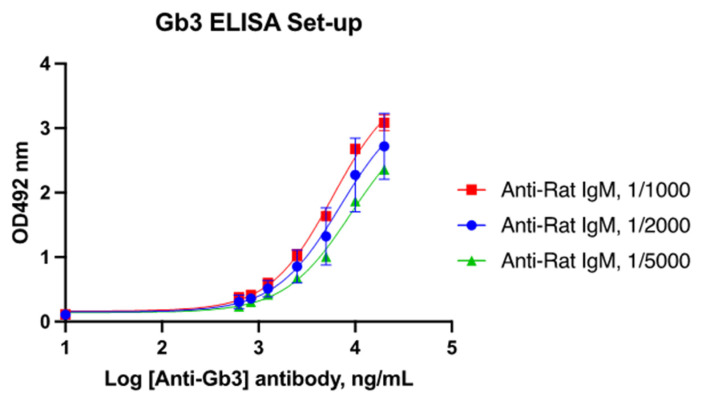
4PL curves generated by using different concentrations of the commercial rat anti-Gb3 antibody (0.600–20 μg/mL), and three dilutions of antirat IgM secondary antibody, as indicated.

For the development of the in-house ELISA, the FSL-Gb3 construct (Sigma Aldrich, Darmstadt, Germany), which is composed of Gb3 conjugated with the function–spacer–lipid (FSL) group, was employed. Different concentrations of FSL-Gb3 (ranging from 1 to 250 μM) were diluted in PBS pH 7.2 and used for the coating of Maxisorp microplates (NUNC). Coating was carried out for 3 h at 25 °C and at 4 °C for 12 h, and then, the plates were washed three times with PBS-T. The plate wells were then blocked with PBS-BSA at room temperature for 1 h. The commercially available anti-CD77 (globotriaosylceramide Gb3) monoclonal antibody produced in rat (GeneTex 2456 Alton Pkwy, Irvine, CA, USA) was used as primary Ab in order to verify the coating efficiency. These preliminary results showed that coating overnight at 4 °C with 5 μM FSL-Gb3 was suitable for assay development. Further adjustments were made according to the results obtained by serial dilutions of the rat anti-Gb3 antibody. Dilutions ranging from 20 μg/mL to 0.6 ng/mL were employed in combination with different dilutions (1/1000, 1/2000, and 11/5000) of the secondary HRP-conjugated antibody specific for rat IgM (Abcam, Cambridge, UK). The assay was considered adequate when the 4PL curve generated plotting OD and antibody concentrations showed linearity (R2) > 0.95. The obtained results indicated with the positive control (rat anti-Gb3 antibody) that the dilution linearity was adequate for all tested conditions, so the coating with 5 μM FSL-Gb3 overnight at 4 °C was employed for assay development (Figure 1).

Human serum from FDCM patients (n = 12) and two pools from healthy individuals (n = 40 pooled samples) were then employed to verify the suitability of the assay for the analysis of human anti-Gb3. After preliminary tests performed with serial dilutions of serum samples (from 1:10 to 800), the dilution 1/50 was selected. 

The samples were prepared in PBS-BSA 1% and added to the wells coated with FSL-Gb3 for 1 h at RT, in duplicate. After washing three times, peroxidase-conjugated goat antihuman Pan Ig (Jackson ImmunoResearch Europe Ltd., Ely, UK) diluted 1:2000 in PBS-BSA 1% was added and the plates were incubated as indicated above, followed by three washes and the addition of OPD substrate (Sigma Aldrich, St. Louis, MO, USA). The plates were then incubated in the dark at RT for 10 min and the reaction was stopped by adding 3N HCl. The plates were read at 492 nm using a microplate reader (BioTek^®^, Winooski, VT, USA). The resulting optical density (OD) measured in the FDCM serum samples was used to calculate the positive/negative (P/N) ratio (Table 1).

The cut-off point was set by analyzing n = 12 FDCM serum samples of FDCM patients with myocarditis and FDCM patients without myocarditis analyzed blindly. A common method to define a cut-off for discriminating positive samples from the negative ones is calculated by applying the following formula: cut-off = average value negative control (pool from 40) + 5 × negative control standard deviation.

According to the analysis, the optimal cut-off value of circulating anti-Gb3 antibody in FDCM patients for predicting myocardial inflammation was P/N > 2.56 fold-change with respect to the reference (or control).

#### 2.2.4. Molecular Study

Definition of virus-negative, immune-mediated, overlapping myocarditis followed the presence at histology of ≥7 CD3+ T lymphocytes per lo- power field associated with focal necrosis of the adjacent myocytes (Figure 5A,B). Negative PCR on two frozen endomyocardial samples for the most common cardiotropic viral genomes including adenovirus, Epstein–Barr virus, herpes virus, parvovirus B19, cytomegalovirus, enterovirus, influenza, and A/B. Serological positivity for antiheart and antimyosin abs was analyzed.

### 2.3. Statistical Analysis

The normal distribution of all continuous variables was checked with visual methods (Q-Q plot and histogram) and a significance test (Kolmogorov–Smirnov normality test and Shapiro–Wilk’s test). For continuous variables, descriptive statistics were provided (number of available observations, mean, and standard deviation), while the median (interquartile range) was used for non-normal data. Categorical data were described as a number (percentage). Baseline demographic and clinical characteristics will be presented in table format. Student’s *t*-test, the χ^2^ test, and the Fisher exact test were used for comparisons; the difference between variables without normal distribution was tested with the Mann–Whitney U test. For all tests, a *p* value less than 0.05 was considered statistically significant. The receiver-operating characteristics (ROC) curve was used to describe the performance and predictive accuracy of circulating anti-GB3 antibody as a biomarker of myocardial inflammation in FDCM. The sensitivity, specificity, and area under the curve (AUC) were calculated, and the cut-off value was determined using the Youden index. All statistical analyses were performed using R statistical analysis software (R 4.3).

## 3. Results

From January 1996 to December 2021, we enrolled eighty-five patients with histological diagnosis of FDCM; forty-eight (56.5%) of them had an overlapping myocardial inflammation with negative PCR for the common cardiotropic viruses (group A). The remaining 37 FDCM patients had no overlapping inflammation at histology (group B). The demographic, clinical, and instrumental characteristics of the two groups are reported in Table 2. There were no differences in terms of age and sex between the two groups. Otherwise, in patients with FDCM and an overlapping myocardial inflammation (group A), a significant increase in LV mass (104.2 ± 5.1 vs. 94.4 ± 5.7 g/m^2^; *p*-value: 0.04) and reduction in LVEF (51.1 ± 4.6 vs. 58.4 ± 5.7%; *p*-value: 0.03) were documented. A more pronounced electrical instability and incidence of heart failure were reported in group A. Regarding systemic manifestations, in group A, an increased incidence of renal disease with lower eGFR and more noncardiac symptoms were revealed.

### 3.1. Circulating Anti-GB3 Antibodies

**Figure 2:**
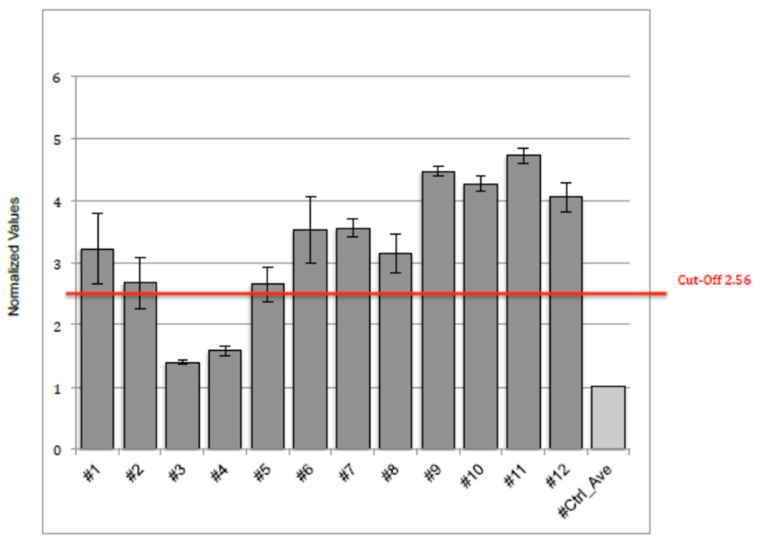
Representative results of n = 2 tests performed with sera diluted 1:50 (2 h at room temperature) and secondary antibodies (1:2000 1 h at room temperature). Each sample was analyzed in duplicate.

The normalized values of the circulating anti-GB3 antibodies assays were performed twice on twelve controls; the values are summarized in Figure 2. As previously described, the calculated cut-off was 2.56. 

The assay results showed that 42 out of 48 FDCM patients with myocarditis were positive for anti-GB3 antibodies, while 30 FDCM patients out of 37 without myocarditis had negative anti-GB3 antibodies (see Table 3).

### 3.2. Cardiac and Myosin Autoantibodies

Those patients with positive anti-Gb3 antibodies had, as previously described (7), serum positivity for antiheart and antimyosin antibodies.

**Figure 3:**
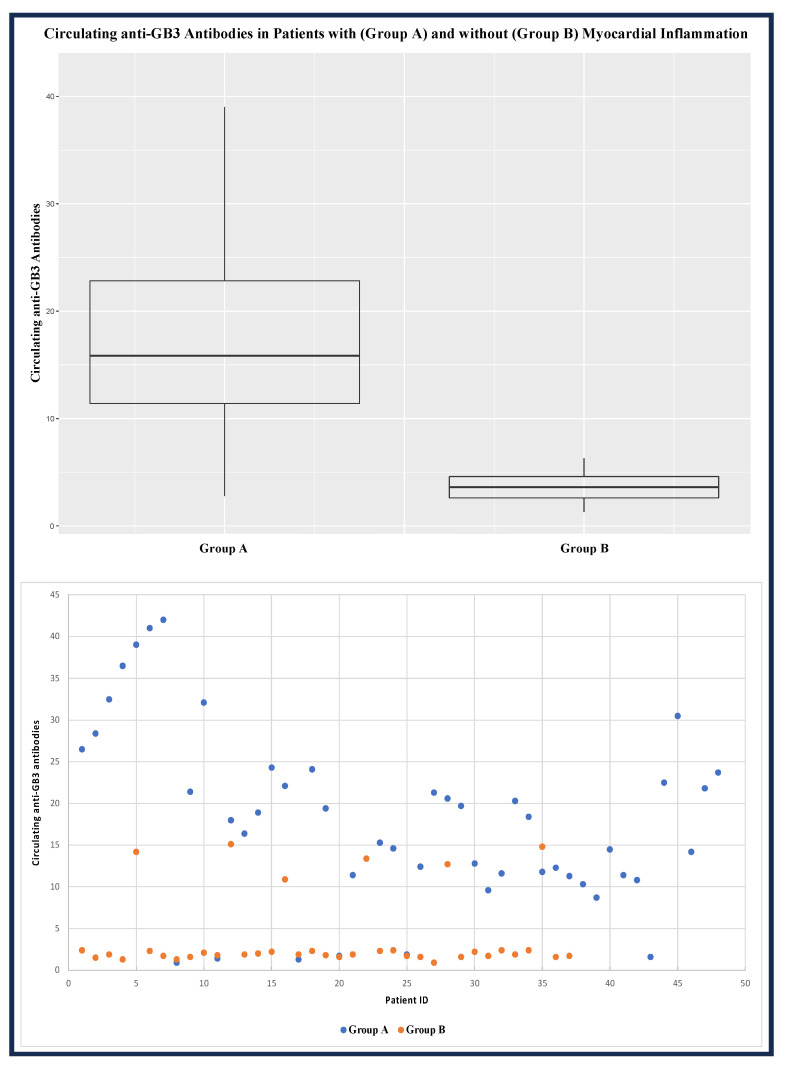
Circulating anti-GB3 antibodies in patients with FDCM with (group A) and without (group B) myocardial inflammation. FDCM: Fabry disease cardiomyopathy; GB3: globotriaosylceramide.

As reported in Figure 3, the value of the circulating anti-GB3 antibodies in group A was significantly higher than in group B [17.2 (IQR: 11.4–22.8) vs. 1.9 (IQR: 1.7–2.4); *p*-value: 0.001].

**Figure 4:**
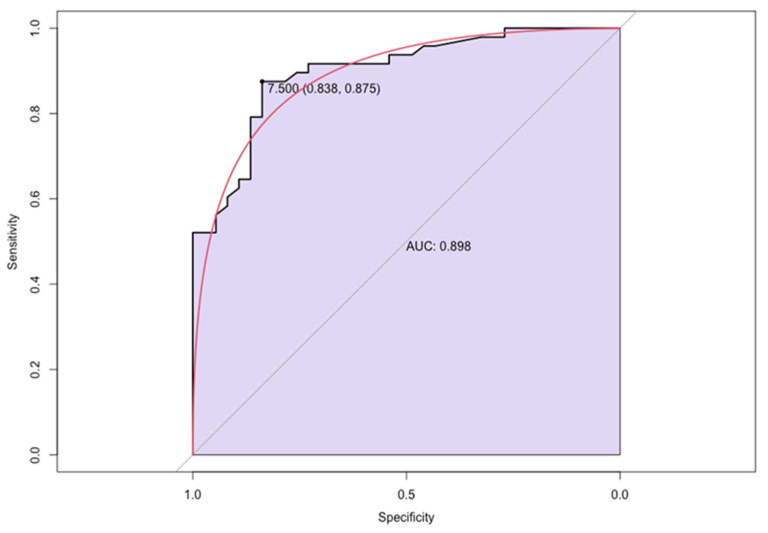
The receiver-operating characteristic curve for describing the performance and predictive accuracy of circulating anti-GB3 antibody as a biomarker of myocardial inflammation in patients with Fabry disease. GB3: globotriaosylceramide.

According to the ROC analysis, the optimal cut-off value of circulating anti-GB3 antibodies in the FDCM patients for predicting myocardial inflammation was >7.5, with a sensitivity of 83.8% and a specificity of 87.5% (the AUC was 0.898, with a 95% confidence interval 0.854–0.942) (Figure 4).

**Figure 5:**
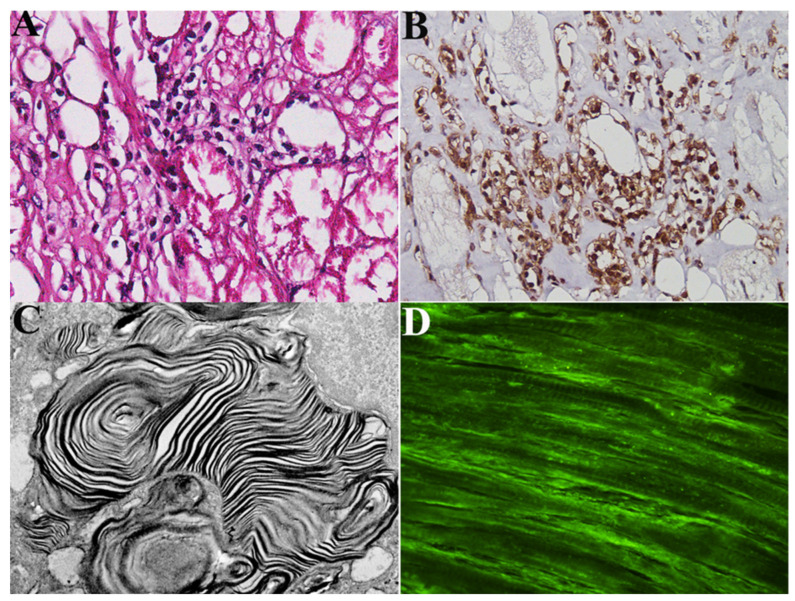
Histology (**A**), immunohistochemistry (**B**), and electron microscopy (**C**) of left ventricular endomyocardial biopsy from a patient with FDCM and overlapping immune-mediated myocarditis. Panel (**D**) shows positive immunofluorescence imaging for antiheart abs. Large cytoplasmic vacuoles into cardiomyocytes (**A**,**B**) exist at ultrastructural examination of myelin bodies (**C**), confirming the diagnosis of FDCM. Myocytes are surrounded by inflammatory infiltrates mainly represented by CD3+ T lymphocytes (**B**) associated with the focal necrosis of adjacent cells. FDCM: Fabry Disease Cardiomyopathy.

All patients with overlapping myocarditis at histology were positive in the serologic assessment of antiheart abs (see Figure 5A–D).

## 4. Discussion

There is growing evidence that myocardial inflammation can be a cause of progression of Fabry disease cardiomyopathy as well as an unnoticed mechanism of disease resistance to ERT. Indeed, myocardial inflammation promotes cell dysfunction and death, causing the expansion of the interstitial space through oedema, inflammatory cell infiltration, and myocardial fibrosis, which result in both ventricular diastolic and systolic dysfunction.

With regard to the origin of myocardial inflammation associated with Fabry disease cardiomyopathy, the absence of viral genomes in the myocardium and the strong positivity of antiheart and antimyosin antibodies described suggest myocardial inflammation to have an autoimmune/disreactive origin [7]. In this regard, GB3, the major component of Fabry disease storage material, is constantly released by the engulfed cardiomyocytes along a constitutional secretory pathway or as a result of cell necrosis. GB3 is recognized as a powerful immunogenic molecule that may promote and perpetuate an inflammatory autoimmune disorder. Indeed, a pro-oxidative and proinflammatory state that correlates with elevated urinary GB3 levels has been described [11,12]. 

The main objective of the present study was the identification of a reliable biomarker of myocardial inflammation overimposing in human FDCM. Indeed, comparing the serologic positivity of anti-GB3 abs with the presence at histology of left ventricular endomyocardial biopsies, the presence of inflammation was confirmed via serology with a sensitivity of 83.8% and a specificity of 87.5%. Remarkably, in our study, the presence of myocardial inflammation in patients with FDCM (group A) was associated with a mean increase in the left ventricular mass index, a lower LVEF, and an increased incidence of atrial fibrillation. Furthermore, in terms of systemic manifestations, a lower mean eGFR and increased noncardiac symptoms were evidenced in group A. These observations suggest myocardial inflammation in FDCM to have a clinical impact impairing cardiac function and increasing electrical instability. Additionally, a systemic compromise such as renal function can occur as well. We are, in fact, unable to attribute a specific pathogenic role to anti-GB3 abs, nor can we establish whether the myocardial damage associated with inflammation should be ascribed exclusively to T lymphocytes. The identification of a sensitive and specific circulating biomarker of cardiac inflammation in patients with FDCM resistant to ERT administration could allow the introduction of immunosuppressive therapy that may halt the progression of the cardiomyopathy and improve the efficacy of ERT. In this regard, the association of anti-GB3 with antiheart and antimyosin abs and negative PCR for the most common viral genomes suggests a strong activation of an immune autoreactive inflammatory process that may benefit from an immune-modulating treatment. A better understanding of the autoimmune/inflammatory activation in Fabry disease and its role in the disease morbidity and mortality will change the therapeutic approach to the disease, allowing the identification of patients with a high autoimmune/inflammatory burden who will benefit from an immunosuppressive treatment in addition to ERT. Positive results of a wider FDCM population are, however, necessary for the implementation of ERT with immunosuppressive therapy. The type of immunosuppressive regimen as well as its duration has yet to be established.

## 5. Conclusions

In conclusion, serologic positivity of anti-GB3 abs represents a reliable biomarker of myocardial inflammation in FDCM. It may suggest ERT implementation with immune-suppressive therapy to enhance ERT impact and improve a patient’s outcome, particularly in those patients with a progressive impairment of cardiac function and/or the occurrence of complex/life-threatening cardiac arrhythmias. Further prospective, double-blind, clinical studies are, however, necessary for the inclusion of immune suppression in the therapeutic regimen of progressive FDCM with inflammation.

## Figures and Tables

**Table 1:** The P/N ratio obtained from 2 independent tests on 12 FDCM serum samples.

	P/N Ratio		
	Test 1	Test 2	Average
#1	3.62	2.82	3.22
#2	2.96	2.38	2.67
#3	1.42	1.37	1.40
#4	1.64	1.53	1.58
#5	2.84	2.45	2.65
#6	3.89	3.14	3.52
#7	3.66	3.45	3.55
#8	2.92	3.37	3.14
#9	4.53	4.41	4.47
#10	4.18	4.36	4.27
#11	4.81	4.63	4.72
#12	4.22	3.89	4.06
#N pool	1.00	1.00	1.00

FDCM: Fabry Disease Cardiomyopathy.

**Table 2:** Baseline clinical, electrocardiographic, and echocardiographic characteristics of the two groups with (A) and without (B) overlapping myocardial inflammation. eGFR: estimated glomerular filtration rate; LVEF: left ventricular ejection fraction.

	Group A (n = 48)	Group B (n = 37)	*p*-Value
Age, yrs	47.6 ± 12.7	48.9 ± 12.5	0.63
Male, n (%)	25 (52.1)	20 (54.1)	0.67
Hypertension, n (%)	6 (12.5)	7 (18.9)	0.55
Heart Failure, n (%)	14 (29.2)	5 (13.5)	0.07
Atrial arrhythmia, n (%)	5 (10.4)	2 (5.4)	0.56
Left ventricular mass index (g/m^2^)	104.2 ± 5.1	94.4 ± 5.7	0.04
LVEF, %	51.1 ± 4.6	58.4 ± 5.7	0.03
Kidney disease, n (%)	8 (16.7)	2 (5.4)	0.18
eGFR	80.4 ± 23.6	89.4 ± 21.3	0.09
Noncardiac symptoms, n (%)	27 (56.2)	15 (40.5)	0.19

**Table 3:** Serum values of GB3 abs in FDCM patients with (Column A) and without evidence of overlapping myocardial inflammation (Column B) at LV endomyocardial biopsy. Compared with normal pooled (n = 40) serum determination (Column C).

Patient ID.	Group		
	A	B	C
1	26.5	2.4	2.56
2	28.4	1.5	
3	32.5	1.9	
4	36.5	1.3	
5	39	14.2	
6	41	2.3	
7	42	1.7	
8	0.9	1.3	
9	21.4	1.6	
10	32.1	2.1	
11	1.4	1.8	
12	18	15.1	
13	16.4	1.9	
14	18.9	2	
15	24.3	2.2	
16	22.1	10.9	
17	1.3	1.9	
18	24.1	2.3	
19	19.4	1.8	
20	1.7	1.6	
21	11.4	1.9	
22	13.4	13.4	
23	15.3	2.3	
24	14.6	2.4	
25	1.9	1.7	
26	12.4	1.6	
27	21.3	0.9	
28	20.6	12.7	
29	19.7	1.6	
30	12.8	2.2	
31	9.6	1.7	
32	11.6	2.4	
33	20.3	1.9	
34	18.4	2.4	
35	11.8	14.8	
36	12.3	1.6	
37	11.3	1.7	
38	10.3		
39	8.7		
40	14.5		
41	11.4		
42	10.8		
43	1.6		
44	22.5		
45	30.5		
46	14.2		
47	21.8		
48	23.7		

## Data Availability

The datasets used and analyzed during the current study are available from the corresponding author upon reasonable request.

## Abbreviations

α-Gal A	Alpha-galactosidase A
AUC	Area under the curve
ERT	Enzyme replacement therapy
FDCM	Fabry disease cardiomyopathy
FSL	Function–spacer–lipid
GB3	Globotriaosylceramide
MSD	Meso Scale Discovery
ROC	Receiver-operating characteristics
